# Development of Coated PLA Films Containing a Commercial Olive Leaf Extract for the Food Packaging Sector

**DOI:** 10.3390/antiox13050519

**Published:** 2024-04-26

**Authors:** Cecilia Fiorentini, Giulia Leni, Elena Díaz de Apodaca, Laura Fernández-de-Castro, Gabriele Rocchetti, Claudia Cortimiglia, Giorgia Spigno, Andrea Bassani

**Affiliations:** 1 Department for Sustainable Food Process (DiSTAS), Università Cattolica del Sacro Cuore, Via Emilia Parmense, 84, 29122 Piacenza, Italygiulia.leni@unicatt.it (G.L.); claudia.cortimiglia@unicatt.it (C.C.); andrea.bassani@unicatt.it (A.B.); 2 TECNALIA, Basque Research and Technology Alliance (BRTA), Parque Tecnológico de Álava, C/Leonardo Da Vinci 11, 01510 Miñano, Álava, Spain; elena.diazdeapodaca@tecnalia.com (E.D.d.A.); laura.fernandezdecastro@tecnalia.com (L.F.-d.-C.); 3 Department of Animal Science, Food and Nutrition (DIANA), Università Cattolica del Sacro Cuore, Via Emilia Parmense, 84, 29122 Piacenza, Italy; gabriele.rocchetti@unicatt.it

**Keywords:** active packaging, antioxidant, metabolomics, phenolic compound, antimicrobial

## Abstract

A commercial olive leaf extract (OL), effective against *Salmonella enterica*, *Escherichia coli*, *Listeria monocytogenes*, and *Staphylococcus aureus*, was added to three different coating formulations (methylcellulose, MC; chitosan, CT; and alginate, ALG) to produce active polylactic acid (PLA) coated films. Evaluation of these coated PLA films revealed significant inhibition of *S. aureus* growth, particularly with the MC and CT formulations exhibiting the highest inhibition rates (99.7%). The coated films were then tested for food contact compatibility with three food simulants (A: 10% ethanol; B: 3% acetic acid; D2: olive oil), selected to assess their suitability for pre-cut hams and ready-to-eat vegetables in relation to overall migration. However, coated films with active functions exhibited migration values in simulants A and B above legal limits, while promising results were obtained for simulant D2, highlighting the need to deeply investigate these coatings’ impact on a real food system. Untargeted metabolomics revealed that the type of coating influenced the selective release of certain phenolic classes based on the food simulant tested. The Oxitest analysis of simulant D2 demonstrated that the MC and ALG-coated PLA films slightly slowed down the oxidation of this food simulant, which is an edible vegetable oil.

## 1. Introduction

In recent years, new food packaging concepts have been introduced to reduce the use of conventional packaging and to satisfy the growing demand for high-quality, ready-to-eat foods that contain few preservatives [1]. Therefore, current innovations in food packaging research are focusing on the development of bio-based and biodegradable packaging that can substitute those made of traditional plastics. In addition, the new trend focuses attention on active materials that incorporate different additives such as nutrients, antioxidants, and antimicrobial substances with the aim of extending the shelf-life of packaged foods [2]. Such active materials can be obtained by different techniques, for example, by incorporating the active agent into or on the contact surface of the material with the packaged food [3].

Among the existing techniques, extrusion is the most widely used procedure to include natural extracts in the final film formulation [3]. However, this technique can often result in thermal degradation of bioactive compounds due to the high temperatures reached. Therefore, natural extracts, or in general, bioactive agents, are preferably produced by a non-heating method, such as surface coating. The latter is a process in which the surface of an object (i.e., onto a food surface or a polymer surface) is coated at low temperature with an active solution through layer-by-layer coating, spray coating, or bar/knife coating technique [4,5].

Some of these methods (e.g., bar coating) have several advantages, such as industrial-scale applicability and a lower probability of affecting the properties of the base polymeric material [3]. In addition, since the active agent is incorporated into the coating layer in direct contact with the food, the use of smaller amounts of active compounds is needed to impart sufficient efficacy [6]. However, given the enhanced release of active agents from coated films to food products, the associated human health risk for food application must be considered. 

Active agents for the production of active coatings include extracts obtained from natural sources, such as agri-food by-products, that are increasing their attention worldwide [7,8]. One example is the olive leaf (OL), a residue abundantly produced by the olive oil industry during tree pruning and olive harvesting, from which it is possible to obtain extracts characterized by both antioxidant and antimicrobial properties due to the phenolic composition (rich in oleuropeosides, flavones, flavonols, flavan-3-ols, and catechin substituted phenols) [9,10]. These extracts have already been demonstrated to be effective for the development of novel active packaging materials [11,12,13,14]. 

Starting from these background conditions, the aim of this study was to characterize, in terms of untargeted polyphenol profile and in vitro bioactivities (both antioxidant capacity and antimicrobial activity), a commercial OL extract. The OL extract was deliberately chosen as the active component for coating formulations intended to be applied onto commercial polylactic acid (PLA) film in order to produce an active film suitable for food packaging purposes. This choice was driven by the well-documented antioxidant and antimicrobial properties of olive leaf extract, attributed to its rich phenolic composition. In this context, the material’s performance and suitability for food contact were assessed through overall migration testing, utilizing three distinct food simulants. Additionally, for the first time, untargeted metabolomics was performed to evaluate the specific migration of phenolic compounds from the PLA films to the food simulants. This approach introduces the novelty of employing untargeted metabolomics to investigate the migration of phenolic compounds from the active coating layer of innovative packaging material to food simulants, representing a pioneering effort in elucidating the specificity of compound release.

## 2. Materials and Methods

### 2.1. Bioactivity of Olive Leaf Extract

A food-grade commercial encapsulated (with maltodextrins) olive leaf powdered extracts was supplied by an Italian company (E.V.R.A.) and stored in the dark until use. Then, to evaluate its total phenolic content (TPC), in vitro antioxidant activity, untargeted phenolic profile, and minimum inhibitory concentration (MIC), the OL extract was solubilized in distilled water and stored at 4 °C until use.

#### 2.1.1. Total Phenolic Content

The TPC of the commercial OL extract (solubilized in distilled water at a concentration of 10 mg/mL) was analyzed using the Folin–Ciocalteu assay, as reported by Bassani et al. [15]. The results were expressed as mg_GAE_/g of extract (GAE, gallic acid equivalent) through a calibration curve obtained with standard gallic acid in water (Fluka, 100–800 mg/L, R^2^ = 0.999).

#### 2.1.2. In Vitro Antioxidant Capacity (FRAP and ABTS Assays)

The ferric-reducing antioxidant capacity (FRAP) of the commercial OL extract (solubilized in distilled water at a concentration of 10 mg/mL) was evaluated according to the procedure described by Bassani et al. [15]. The results were expressed as µmol_Fe(II)/_g of extract using a calibration curve with FeSO_4_·7H_2_O in water (Carlo Erba, Milan, Italy; 0.2–2 mmol_Fe(II)/_L, R^2^ = 0.997). In addition, the ability of the extract to reduce the 2,2′-azino-bis(3-ethylbenzothiazoline-6-sulfonic acid (ABTS) radical was assessed according to the method of Bassani et al. [15]. The calibration curve was obtained with a Trolox^®^ standard (Tr) in 50% of ethanol (Sigma-Aldrich, Milan, Italy; 100–500 mg_Tr_/L, R^2^ = 0.999), and then the results were calculated and expressed as μmol_Tr_/g of extract.

#### 2.1.3. Untargeted Phenolic Profiling by UHPLC-HRMS Approach

High-resolution mass spectrometry (HRMS; Thermo Scientific, Waltham, MA, USA) was used to perform an untargeted analysis. The latter allowed us to putatively identify the phenolic compounds characterizing the OL extract and food simulants A and B after migration from the coated PLA films. The untargeted analysis was conducted as reported by Fiorentini et al. [16]. Ferulic acid (phenolic acids), quercetin (flavonols), catechin (flavanols), cyanidin (anthocyanins), luteolin (flavones and other flavonoids), resveratrol (stilbenes), and oleuropein (for tyrosol derivatives and lower-molecular-weight compounds) were used as qualitative and quantitative standards of their respective classes. Then, after having assessed the cumulative phenolic equivalent concentration, the active compound migration was evaluated. Based on the concentration of the commercial OL extract into the coating formulation (<50 mg/mL), the volume of the coated solution used for each coated PLA film (about 2 mL for 3.75 dm^2^ surface), and the ratio between the coated PLA film and food simulants used for the migration tests (0.36 dm^2^ in 36 mL), the maximum extract concentration that could potentially migrate into food simulants was evaluated. Moreover, the greatest concentration of the total equivalent phenolics that could migrate and the fractions of the migrated compounds were evaluated following the method reported by Fiorentini et al. [16].

#### 2.1.4. Antimicrobial Activity

The MIC of the OL extract was assessed based on what was reported by Fiorentini et al., testing *Escherichia coli* ATCC 25922 and *Salmonella enterica* serovar Typhimurium DSM 17058, *Listeria monocytogenes* DSM 15675 and *Staphylococcus aureus* ATCC 33591 [16]. Briefly, fresh plate cultures were suspended in a sterile saline solution to achieve a turbidity of 0.5 McFarland. Subsequently, this bacterial suspension was diluted 1:1000 to serve as the inoculum. In 96-well microtiter plates, 100 μL of double-concentrated culture medium, 50 μL of diluted extract, and 50 μL of bacterial inoculum were added to each well, and then the plates were incubated in triplicate at 37 °C for 24 h. Finally, growth was monitored at 620 nm using a Multiskan Microplate Spectrophotometer (Themo Fisher Scientific, Waltham, MA, USA). Results were reported as the Minimum Inhibitory Concentration (MIC), representing the lowest extract concentration (mg/mL) capable of inhibiting microbial growth.

### 2.2. Coating Preparation

Three different types of coating formulation were produced using chitosan (CT) (Food grade, MW 169 kDa and deacetylation degree of 84%, Trades, S.A., Barcelona, Spain), alginate (ALG) (Manugel GMB from FMC Biopolymer, UK), and methylcellulose (MC) (E-461, Epsa, Torrent, Spain). The CT formulation was prepared as 1% wt. chitosan solution in a 1% acetic acid aqueous solution. The chitosan solution was stirred at room temperature until it was completely dissolved. The aqueous solution of ALG was obtained at 2% wt. by dispersing the powder in distilled water at room temperature under stirring. The MC formulation was prepared by dispersing and solubilizing 1.5% wt. of methylcellulose in distilled water at 80 °C with magnetic stirring and then allowed to cool to room temperature. In all formulations, the OL extract was then added at a concentration < 50 mg/mL. Then, each prepared coating solution (i.e., CT + OL, MC + OL, and ALG + OL) was coated onto PLA films (INGEO^TM^ PLA, Natureworks, Minneapolis, MN, USA) using the surface coating technique. In particular, the PLA film was mounted on the coater surface, and 2–3 mL of each coating solution was spread over the film using a lab bar coater (RK-101 Control Coater, RK Print Coat Instruments, Royston, The United Kingdom). All the coated PLA films were dried at room temperature for 3 h and lastly stored at 4 °C in the dark until used.

### 2.3. Migration Test

#### 2.3.1. Overall Migration

The overall migration (M) test was performed with three different food simulants, as reported by Fiorentini et al. [16], with some modifications. The selected food simulants were 10% ethanol (*v*/*v*), 3% acetic acid (*w*/*v*), and olive oil (i.e., simulants A, B, and D2, respectively) and the contact conditions 10 days at 40 °C. Furthermore, specimens of 0.36 dm^2^ of each coated PLA film were cut and wrapped to obtain a pouch of 0.36 dm^2^ with the coating side facing outward (reverse pouch) in order to check migration only from the coated side. The PLA film represented the control sample for the blank correction and was analyzed to check the initial contamination. All specimens obtained were then completely immersed in 36 mL of each simulant, as stated by the European Standards EN-1186 [17]. 

#### 2.3.2. Migration of Bioactive Compounds

The detection of subclasses of migrated phenolic compounds (UHPLC-HMRS) was evaluated following a 10-day exposure at 40 °C on simulants A and B, as described in Section 2.1.3. For simulant D2, an indirect method was applied to measure the efficiency of the coating in providing antioxidant protection to fatty foods. Therefore, after the contact, D2 simulant-coated films, Oxitest analysis (Oxitest instrument, Velp-Scientifica, Deer Park, NY, USA) was directly performed on D2 simulant as reported by Fiorentini et al. [16]. Briefly, the Oxitest analysis was conducted using 10 g of food simulant D2, and the measurements were taken immediately (within a few seconds) after 10 days of exposure at 40 °C. An Oxitest was set to operate at 100 °C with an initial pure oxygen pressure of 6 bar. The consumption of oxygen was tracked by monitoring changes in absolute pressure within the instrument chambers over time. Subsequently, the resulting oxidation curve was analyzed using OXI SoftTM software 3.0.0 (Velp-Scientifica, Varese, Italy) to determine the induction period (IP). As a control, a sample of olive oil was subjected to the same environmental conditions without contacting the film during the experiment.

### 2.4. Antimicrobial Activity of Coatings

The method ISO 22196:2011 [18] was used to evaluate both the antimicrobial activity and the potential antimicrobial effect of all the coated PLA films against *S. aureus* ATCC 6538P. The bacterial suspension presented a concentration of 3.5 × 10^6^ CFU/mL in a nutrient broth medium (Oxoid Ltd., Hampshire, UK). The results were elaborated to calculate the antibacterial activity (R), which is the number of decimal reductions observed on the coated film corrected by the decimal reductions observed on the untreated film. The percentage growth inhibition can then be calculated from R as (1–10^−R^) × 100. 

### 2.5. Statistical Analysis

These collected data were subjected to ANOVA followed by Tukey’s post hoc test to determine the differences (*p* < 0.05) between samples using IMB SPSS Statistics (Version 25). The experiments were carried out in triplicate and are reported as mean ± standard deviation values. Regarding the phenolic profiling, an unsupervised principal component analysis (PCA) followed by orthogonal projections to latent structures discriminant analysis (OPLS-DA) was carried out using the software MetaboAnalyst (Version 5.0) [19] considering all the phenolic compounds putatively annotated by UHPLC-HRMS. The OPLS-DA model validation parameters, namely goodness-of-fit (R^2^Y) and goodness-of-prediction (Q^2^Y) were also inspected. Additionally, the significance of each compound for discrimination purposes was assessed using the variable’s importance in the projection (VIP) selection method. The discriminant compounds that have a VIP score greater than 1 were considered the most significant in the prediction model, as previously reported [20]. All the analyses were performed considering the food simulant and the type of coating formulation.

## 3. Results

### 3.1. Bioactivity of Olive Leaf Extract

#### 3.1.1. Total Phenolic Content and Antioxidant Capacity

The concentration of TPC in OL extract, according to the Folin assay, was equal to 54.24 ± 2.00 mg_GAE_/g_dm_. Overall, a greater content of polyphenols in plant extracts corresponds to a higher antioxidant capacity [21]. Making comparisons between our data and those presented in the scientific literature posed a challenge due to our lack of knowledge about the extraction method used, including any purification steps used by the company from which the extract was purchased. In addition, the yield and composition of phenols originating from different sources were found to vary depending on the genetic background of the plant and extraction techniques [22]. Cifà et al. determined for OL extracts obtained with techniques such as microwave-assisted extraction, steam explosion, and pressurized liquid extraction, a value of TPC ranging from 0.0025 mg_GAE_/g_dm_ to 144.2 mg_GAE_/g_dm_ [23]. Giacometti et al. found an average TPC value of 59.03 ± 3.14 mg_GAE_/g_dm_ in OL extract obtained with 80% ethanol by conventional extraction procedure [24]. Other authors obtained comparable values of TPC from olive leaves subjected to extractions with heated 9% glycerol and pressurized liquid, respectively [25,26].

OL represents an organic substrate rich in total phenols and with potentially antioxidant properties. In the present work, the OL extract antioxidant capacity was in vitro measured by the ABTS and FRAP assays, obtaining values of 851.16 ± 34.27 µmol_Fe(II)_/g_dm_ and 242.16 ± 10.19 µmol_Tr_/g_dm_, respectively. Similar results were obtained by Giacometti et al., who subjected OL to conventional and ultrasound-assisted extractions, obtaining extracts with 258.82 and 295.80 µmolTr/g_dm_ by ABTS assay [24]. Ghasemi et al. obtained extracts from Iranian olive (by percolation method with MeOH/H_2_O 50/50) with antioxidant capacity which ranged from 358.66 ± 0.004 µmol_FeII_/g dried extract to 1971.37  ± 0.007 µmol_FeII_/g dried extract [27].

#### 3.1.2. Untargeted Phenolic Profiling by UHPLC-HRMS Approach

The profile of polyphenols that characterized the commercial OL extract under investigation was evaluated through the UHPLC-HRMS analysis and is reported in Table S1 of Supplementary Materials. The analysis allowed the potential identification of 151 compounds against the comprehensive databases Phenol-Explorer and FoodDB, which are primarily made up of flavonoids, phenolic acids, tyrosol-derivates, lower-molecular-weight phenolics, and stilbenes. Moreover, the QCs allowed us to structurally confirm the identity of 92 compounds (namely 39 flavonoids, followed by 26 other phenolics, 25 phenolic acids, and 2 stilbenes). 

The most abundant class was that represented by “other phenols,” including several tyrosol-related metabolites with a total concentration of 1470.78 ± 155.66 µg/L (expressed as oleuropein equivalent) (Table S2 of Supplementary Materials). The extract included oleuropein and its aglycone, as also reported by many authors [28,29,30], together with ligstroside-aglycone, demethyloleuropein, and 3,4-DHPEA-EA (i.e., elenolic acid mono-aldehyde), already known as the principal compounds of olive leaves. Oleuropein, which is naturally present in olive trees and its by-products, belongs to a specific group of coumarin-like compounds called secoiridoids, and it is known to have different bioactive properties [10]. The second most abundant class revealed by untargeted phenolic profiling was phenolic acids that have a concentration of 700.35 ± 201.96 µg/L, expressed as ferulic acid equivalent. Additionally, the OL extract under investigation showed a notable presence of flavonoids, found both in the glycosylated (e.g., luteolin 7-*O*-(2-apiosyl-6-malonyl)-glucoside, luteolin 7-*O*-glucoside, luteolin 7-*O*-malonyl-glucoside, luteolin 7-*O*-rutinoside, and kaempferol 7-*O*-glucoside) and the aglycone form (e.g., apigenin, luteolin, and catechin). Nevertheless, it is worth taking into consideration that the phenolic composition of olive by-products is strictly affected by the cultivar, the environmental conditions, the ripening level, technological factors, and industrial processes used for extraction [9,24,28,31].

#### 3.1.3. Antimicrobial Activity

OL extract antimicrobial activity was evaluated against four different pathogens that can often contaminate food products, including both Gram-negative and Gram-positive microorganisms. The commercial OL extract demonstrated antimicrobial properties with respect to all tested microorganisms, with MIC values that ranged from 1.25 mg/mL for the *S. aureus* test to 5 mg/mL for *L. monocytogenes* and 10 mg/mL for both *E. coli* and *S. enterica* trials.

These results were in agreement with several authors who have reported that OL extract exhibits inhibitory activities against *E. coli*, *S. aureus*, and *L. monocytogenes* [30,32]. Furthermore, these data are consistent with the ones reported by Giacometti et al., in which OL extract, obtained with the conventional extraction method, appears to be more efficient in inhibiting *S. aureus* (MIC 2.00 ± 0.80 mg/mL) than *L. monocytogenes* (MIC 5.60 ± 1.60 mg/mL) and *E. coli* (MIC 8.00 ± 3.20 mg/mL) [24]. 

Therefore, since the commercial OL extract demonstrated good antimicrobial properties towards all the microorganisms tested, food packaging with antimicrobial properties could be produced with such extracts in order to increase the shelf-life of the packed foods. 

### 3.2. Migration Test

#### 3.2.1. Overall Migration

This test allowed us to determine the maximum amount of substances that can be released from the polymeric material to the food packed, and that should not exceed 10 mg/dm^2^ (Reg. EU 10/2011). The three food simulants A, B, and D1, in combination with the contact conditions of 10 days at 40 °C, were evaluated in accordance with Reg. EU 10/2011. This assessment aimed to establish their compliance with the overall migration limit applicable for all types of foods for “any long-term storage at room temperature or below, including when packaged under hot-fill conditions, and/or heating up to a temperature T where 70 °C ≤ T ≤ 100 °C for a maximum of t = 120/2 × ((T − 70)/10) minutes”. This choice was taken in the context of the research project during which the new materials were developed and which foresaw testing potential use for pre-cut hams and ready-to-eat vegetables. The results are reported below in Table 1.

In particular, the overall migration ranged from 0.56 ± 0.48 mg/dm^2^ for the uncoated PLA into simulant A to 44.24 ± 2.31 mg/dm^2^ for the ALG coating into simulant B. Only five trials showed the overall migration levels below the legal limit. Since the tested samples were coated films with an active function and this property is not an intrinsic characteristic of the passive material, the amount of active substance released should not be calculated in the overall migration value (Regulation No 450/2009, p. 2), and the corrected migration limit could be lower than the measured ones. Furthermore, food-grade components were used for the coating preparation, but it is evident that with simulants A and B, most of the coating would be released on the food product, and this would lead to a different application than what was originally planned. 

The uncoated PLA resulted in compliance with the regulations regarding overall migration for the tested conditions. In addition, positive results were obtained also for PLA coated with CT and MC with simulant D2. The lower migration of compounds in the case of simulant D2 was partially expected since the coating layers were developed as water-soluble formulations. Moreover, the migration values determined in simulants D2 could be lower. In fact, Regulation (EU) No 10/2011 reports that the overall migration might be reduced from 2 to 5 times, depending on the fatty foods other than pure fats.

#### 3.2.2. Migration of Bioactive Compounds and Multivariate Statistical Discrimination

The untargeted metabolomics was also performed to identify the phenolic compounds that migrated from the different coated PLA films to food simulants A and B. Results are reported below in Table 2. 

The phenolic classes reported almost the same trend of migration in all samples for each food simulant, except for flavonols, stilbenes, and tyrosol derivatives, for which it was detected variations among samples were higher than 20%. In general, CT was the film that provided the highest rate of migration for all phenolic classes, except for stilbenes in food simulant A. On the contrary, stilbenes presented the highest concentration in the MC sample. 

The cumulative concentration of total phenolic equivalents was evaluated for both the commercial OL extract and each extract that migrated into the food simulants (Table 3).

The total phenol concentration that migrated from the food simulants to the different PLA films ranged from 10.1 ± 1.0 mg Eq./L to 23.2 ± 1.8 mg Eq./L. A higher migration value was obtained for the CT sample, while lower values were calculated for the MC and ALG samples. 

Starting from the content of total phenolic equivalents, it was possible to calculate the percentage of active compounds that transferred from the PLA film to the simulant. In fact, the PLA films were treated with the extract at a ratio of 0.533 mL/dm^2^, while the contact ratio for the migration test was 100 mL/dm^2^. It was then possible to estimate the maximum achievable concentration of extract (0.2133 mg/mL) and of total equivalent phenolics (49.74 ± 7.83 mg Eq./L) in the simulant. These values come from the assumption of 100% migration, which is obviously an overestimation since migration can proceed to maximum levels until equilibrium conditions are reached. This agrees with the fact that the theoretical values were always higher than the measured ones.

**Figure 1:**
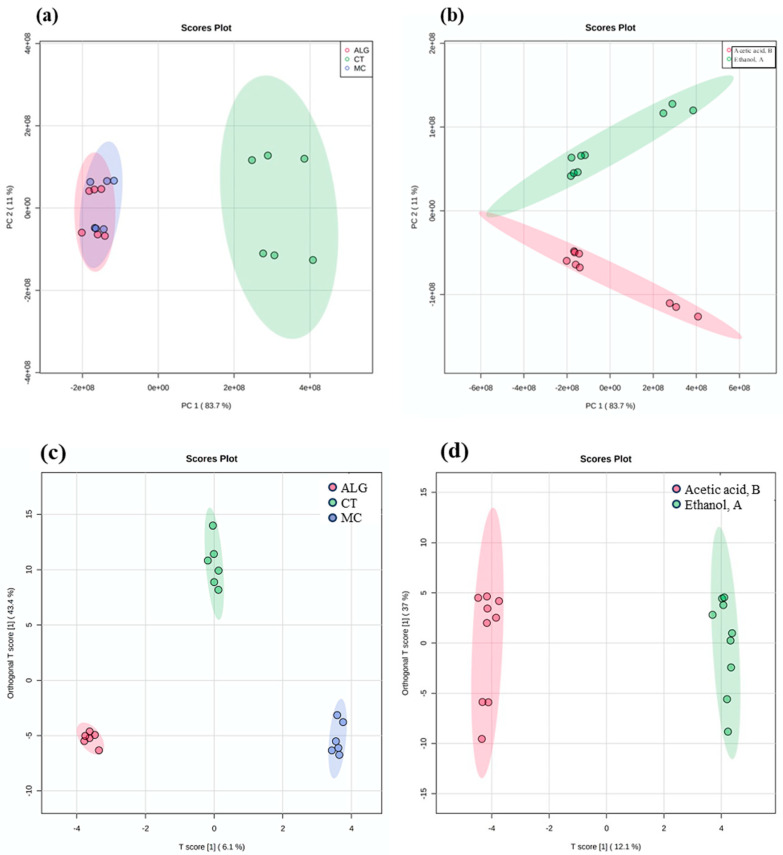
Unsupervised principal component analysis (PCA) considering the profile of migrated polyphenols based on (**a**) the type of PLA film coated (ALG: alginate; CT: chitosan; and MC: methylcellulose) and (**b**) the type of food simulant used (A and B). Supervised orthogonal projection to latent structures discriminant analysis (OPLS-DA) considering the profile of migrated polyphenols based on (**c**) the type of PLA film coated (ALG, CT, and MC), and (**d**) the type of food simulant used (A and B).

Then, an unsupervised PCA analysis was assessed to understand the differences imposed by the migrated compounds between the different samples (Figure 1a,b). 

The score plots revealed a clear discrimination when considering both types of coated PLA film under investigation and the food simulant (Figure 1a and Figure 1b, respectively). In this case, the principal components PC1 and PC2 were found to explain 83.7% and 11%, respectively, of the total variability. Interestingly, the PCA score plot showed a different chemical behavior of the CT sample regardless of the food simulant upon which it is placed in contact (Figure 1a), thus confirming the results reported in Table 3. Overall, this result may be explained by a higher solubility of the CT coating in contact with food simulants compared with the other two samples (ALG and MC), which showed similar behavior. Moreover, the PCA score plot allowed us to observe a clear distinction between the two food simulants, regardless of the type of coating tested (Figure 1b). 

Starting from these unsupervised conditions, a supervised OPLS-DA approach was then performed to highlight the contribution of each major group of discriminating polyphenols (Figure 1c,d) to the output observed. The derived prediction models showed good separation between the different analyzed samples, and we found that the type of coating affected the selective release of some phenolic classes, regardless of the food simulant (Figure 1c). However, the model also provided a clear discrimination between the two food simulants, regardless of the type of coating (Figure 1d). As could have been expected, the phenolic profile of the extract fraction that could migrate into a food matrix is significantly affected by the nature of the food matrix (then by the simulant in our study) due to different affinity between different phenolic compounds and different food components. 

Regarding the goodness parameters of both OPLS-DA models, they showed Q^2^(cum) prediction abilities equal to 0.77 and 0.91, respectively, as well as good correlations values (i.e., R^2^X(cum) = 0.67, R^2^Y(cum) = 0.99 and R^2^X(cum) = 0.61 and R^2^Y(cum) = 0.99, respectively). Based on this, the most discriminating phenolic compounds migrated from the different coated PLA films to the two food simulants were selected with the VIP approach and provided in Figure S1. The VIP scores analysis, based on coating type and selecting compounds with a VIP major than 1 (overall 34 discriminant compounds), showed that the class of phenolic acids was the most discriminant for the comparison ALG vs. CT and MC (Figure S1a). For the CT sample, the most discriminant class of compounds appeared to be tyrosol derivatives. Finally, the MC sample showed an equal distribution of both phenolic classes.

The second VIP score analysis assessed the impact of the simulant on the profile of phenolic compounds, thus allowing the extrapolation of 49 discriminant compounds with a VIP score > 1 (Figure S1b). This approach showed that compounds belonging to both phenolic acids and tyrosol derivatives classes were equally distributed between the two food simulants. However, oleuropein was found to significantly discriminate the simulant A, with a VIP score greater than 2. Therefore, this compound, known for its bioactive properties [10], has a higher affinity with simulant A compared with simulant B, and thus, the coated PLA-based films might be more “active” in contact with non-acidic aqueous foods. Additionally, we found a higher number of discriminating compounds in simulant A belonging to the phenolic class of flavones and flavonoids, including luteolin, known for its antimicrobial properties against *L. monocytogenes* and *S. aureus* [33].

**Figure 2:**
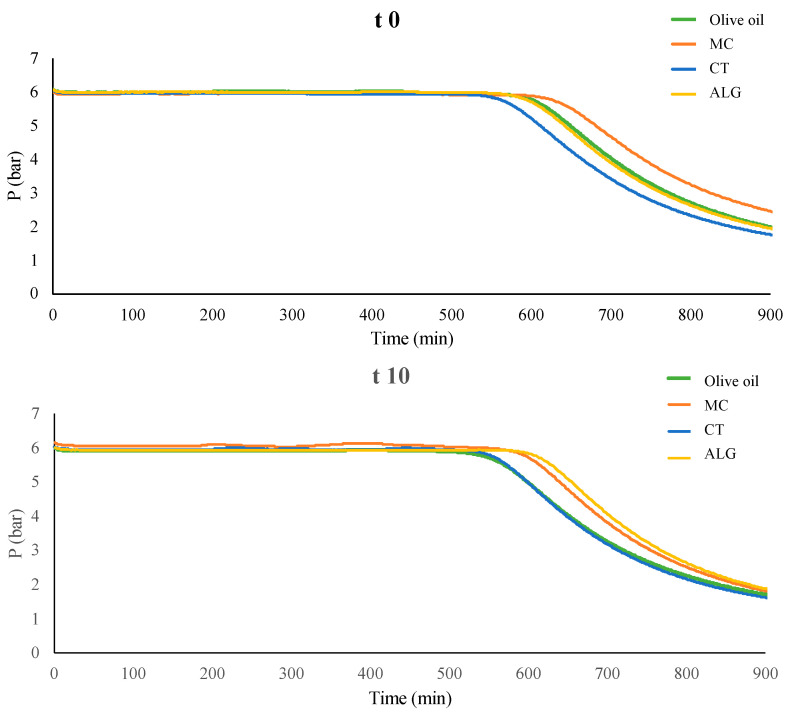
Oxitest analysis after 0 (t 0) and 10 (t 10) days of contact between the different coated PLA films: MC (methylcellulose, orange), CT (chitosan, blue), and ALG (alginate, yellow) and the food simulant D2 (olive oil) (green).

The Oxitest assay was carried out on simulant D2 (oil) to assess if the oxidation could be decreased and/or delayed by the active compounds released into the food simulant. The analysis provides the induction period (IP), calculated as the time on the graph when the pressure line begins to fall. Higher IP is then related to a greater resistance to oxidation over time. After 10 days, the MC and ALG samples seem to differ slightly more from the CT sample and the control (olive oil) (Figure 2).

CT and control samples showed a slightly shorter IP than the other samples. Since a shorter IP indicates a lower oxidative stability, the films coated with MC and ALG seemed to slightly increase the oil stability, which would indicate a potential release of antioxidant compounds from the coating layer to this food simulant.

Since the migration rate is also influenced by different factors, including food composition, a_w_, pH, and the humidity level in the storage environment, which are not fully accounted for with food simulants [34], these aspects should be considered in future research.

### 3.3. Antimicrobial Activity

Since the OL extract had shown the lowest MIC value against *S. aureus*, the antimicrobial properties of the different coated films were evaluated against this microorganism. The antimicrobial activity R resulted in 2.86, 2.85, and 0.91 for the MC, CT, and ALG-coated samples, respectively. These correspond to a growth percentage inhibition of 99.86% for MC and CT and 87.70% for ALG. It can then be commented that all the coated PLA films almost completely inhibited the *S. aureus* growth, confirming the inhibitory efficacy already demonstrated by the MIC assay. Therefore, these coated films could potentially improve the shelf-life of the food packed thanks to the slowdown of microbial growth.

## 4. Conclusions

The analyzed commercial OL extract showed antimicrobial activity against the microorganisms tested, showing the lowest MIC value against *S. aureus*. The coating formulations (MC, CT, and ALG), containing the OL extract and coated on PLA films, showed values of overall migration above the legal limit of 10 mg/dm^2^ for simulant A and B (simulating hydrophilic foods and hydrophilic foods with pH lower than 4.5, respectively), but not for simulant D2 (simulating lipophilic foods which contain free fats at the surface). Even though the migration values should be corrected by the amount of released active substance according to EU legislation (Reg. 450/2009, p. 2), the measured value indicated a solubilization of most of the coating layer. Even though the coating formulations were developed as potentially edible, this would give a very different application result. All the tested coated PLA films almost completely inhibited the growth of *S. aureus*, confirming the inhibitory efficacy of the OL extract determined in the MIC assay, and among the coated films tested, the MC and CT ones showed the highest inhibition values (around 99.7%). The most abundant class of phenolic compounds characterizing the OL extract was represented by “other phenols” (1470.78 ± 155.66 µg of oleuropein Eq./L). The evaluation of migrated compounds resulted in an overall release of 20 to 46% in food simulants A and B, with the highest values found for the CT-based formulation. Based on the unsupervised PCA analysis, different chemical behaviors of the CT-coated film samples were observed, regardless of the food simulant on which it was placed in contact. The supervised OPLS-DA approach revealed that the type of coating affected the selective release of certain phenolic classes based on the food simulant on which the coated PLA films were placed in contact; from the VIP approach, oleuropein was found to significantly discriminate simulant A. Interestingly, the nontarget metabolomics approach revealed which phenolic classes migrate based on simulant and based on coating. Understanding these relationships enhances the investigation into the migration of natural antioxidant extracts incorporated into packaging, indicating the necessity to recognize that the migration phenomenon can alter the relationships between the initially present phenolic compounds based on the variables involved. In addition, from the Oxitest analysis conducted on simulant D2, the MC and ALG-coated PLA films slightly slowed down the oxidation of this food simulant, which would indicate a possible release of antioxidant compounds from the coating layer to this food simulant and then a potential antioxidant activity for foods with a lipophilic character.

These observations underscore the imperative for thorough investigations into the release kinetics and effectiveness of antioxidant compounds from the coated films within real-world food systems. Understanding how these films interact with diverse food matrices, including perishable items and ready-to-eat products, is pivotal for gauging their impact on extending food shelf-life. Moreover, broadening the scope of antimicrobial testing to encompass a wider array of pathogens relevant to food safety would bolster the applicability of the findings. In addition, there exists a critical need to evaluate the mechanical properties and biodegradability of these materials to assess their environmental footprint comprehensively. Therefore, future research endeavors should prioritize investigating these interactions in conjunction with biodegradation to ensure the efficacy, safety, and environmental sustainability of coated PLA films across diverse food environments.

## Figures and Tables

**Table 1:** Overall migration values obtained for different coated PLA films and from control uncoated PLA.

Coating Formulation	Overall Migration (mg/dm^2^)		
	Simulant A	Simulant B	Simulant D2
Chitosan	34.31 ± 3.44 ^aA^	34.93 ± 1.86 ^aA^	4.31 ± 0.36 ^bA^
Alginate	36.32 ± 5.41 ^aA^	44.24 ± 2.31 ^bB^	11.20 ± 3.26 ^cB^
Metylcellulose	37.07 ± 2.72 ^aA^	30.90 ± 1.39 ^bC^	9.86 ± 2.11 ^cB^
Control (uncoated)	0.56 ± 0.48 ^aB^	3.19 ± 0.73 ^bD^	2.01 ± 0.27 ^cA^

Values reported as mean ± standard deviation. Different lowercase letters were used to indicate significant differences between different food simulants for the same sample, and different uppercase letters were used to indicate significant differences between samples using the same food simulant.

**Table 2:** Evaluation of total phenolic equivalent concentration for the food simulants A and B after the migration of active compounds from different coated PLA films.

Food Simulant	Sample	Phenolic Class Equivalent (µg Std Eq./L)						
		Anthocyanins	Flavones and Flavonoids	Flavanols	Flavonols	Phenolic Acids	Stilbenes	Tyrosol Derivatives
A	MC	197.6 ± 35.8	484.9 ± 86.7	571.5 ± 30.6	31.4 ± 2.8	1588.6 ± 151.7	89.3 ± 2.7	9862.3 ± 722.3
	CT	364.6 ± 19.3	935.9 ± 58.2	662.8 ± 45.2	66.7 ± 4.7	2756.8 ± 150.5	115.2 ± 19.6	18,337.2 ± 1659.2
	ALG	226.1 ± 10.5	439.5 ± 9.1	243.1 ± 22.4	29.1 ± 2.1	1354.6 ± 190.0	91.8 ± 5.3	8732.5 ± 207.4
B	MC	197.5 ± 28.2	463.5 ± 23.2	336.5 ± 54.6	32.8 ± 4.2	1611.5 ± 167.1	120.9 ± 6.8	7702.8 ± 333.1
	CT	376.4 ± 29.2	976.0 ± 52.3	553.9 ± 133.4	68.3 ± 12.9	2286.7 ± 91.4	106.3 ± 14.1	16,816.5 ± 709.0
	ALG	240.8 ± 30.4	449.9 ± 43.6	308.1 ± 30.0	27.1 ± 1.5	1335.0 ± 90.6	102.8 ± 11.4	7635.3 ± 810.9

Values reported as mean ± standard deviation. MC: methylcellulose; CT: chitosan; ALG: alginate.

**Table 3:** Percentage of active compounds migrated from the different coated PLA films in both simulant A and B.

Food Simulant	Coated PLA Film	Total Phenolic Eq. Conc. [mg Eq./L]	Migrated Active Compounds [%]
A	Methylcellulose	12.8 ± 0.7	25.79 ± 1.51 ^aA^
	Chitosan	23.2 ± 1.8	46.72 ± 3.61 ^bB^
	Alginate	11.1 ± 0.2	22.35 ± 0.44 ^aA^
B	Metylcellulose	10.5 ± 0.6	21.04 ± 1.12 ^aA^
	Chitosan	21.2 ± 0.9	42.59 ± 1.86 ^bB^
	Alginate	10.1 ± 1.0	20.30 ± 1.96 ^aA^

Values reported as mean ± standard deviation. Lowercase letters indicate a comparison between samples with the same food simulant, while different capital letters indicate a comparison between samples regardless of the food simulant used.

## Data Availability

The original contributions presented in the study are included in the article/Supplementary Materials; further inquiries can be directed to the corresponding author.

## Supplementary Materials

The following supporting information can be downloaded at https://www.mdpi.com/article/10.3390/antiox13050519/s1. Figure S1: The VIP scores are based on the type of PLA-coated film and the type of food simulant used. Table S1: the metabolomics dataset with each compound together with phenolic class, isotopic MS and MS/MS profiles, and raw abundance values. Table S2: the phenolic equivalent concentrations of the compounds identified in the OL extract by metabolic analysis.
