# Supplementary material for: Development of Coated PLA Films Containing a Commercial Olive Leaf Extract for the Food Packaging Sector
Source: Antioxidants (Basel). 2024 Apr 26;13(5):519. doi: 10.3390/antiox13050519 (PMC11117849; doi:10.3390/antiox13050519)
Supplement: Supplementary file 1 [file antioxidants-13-00519-s001.zip › Figure S1.pdf]

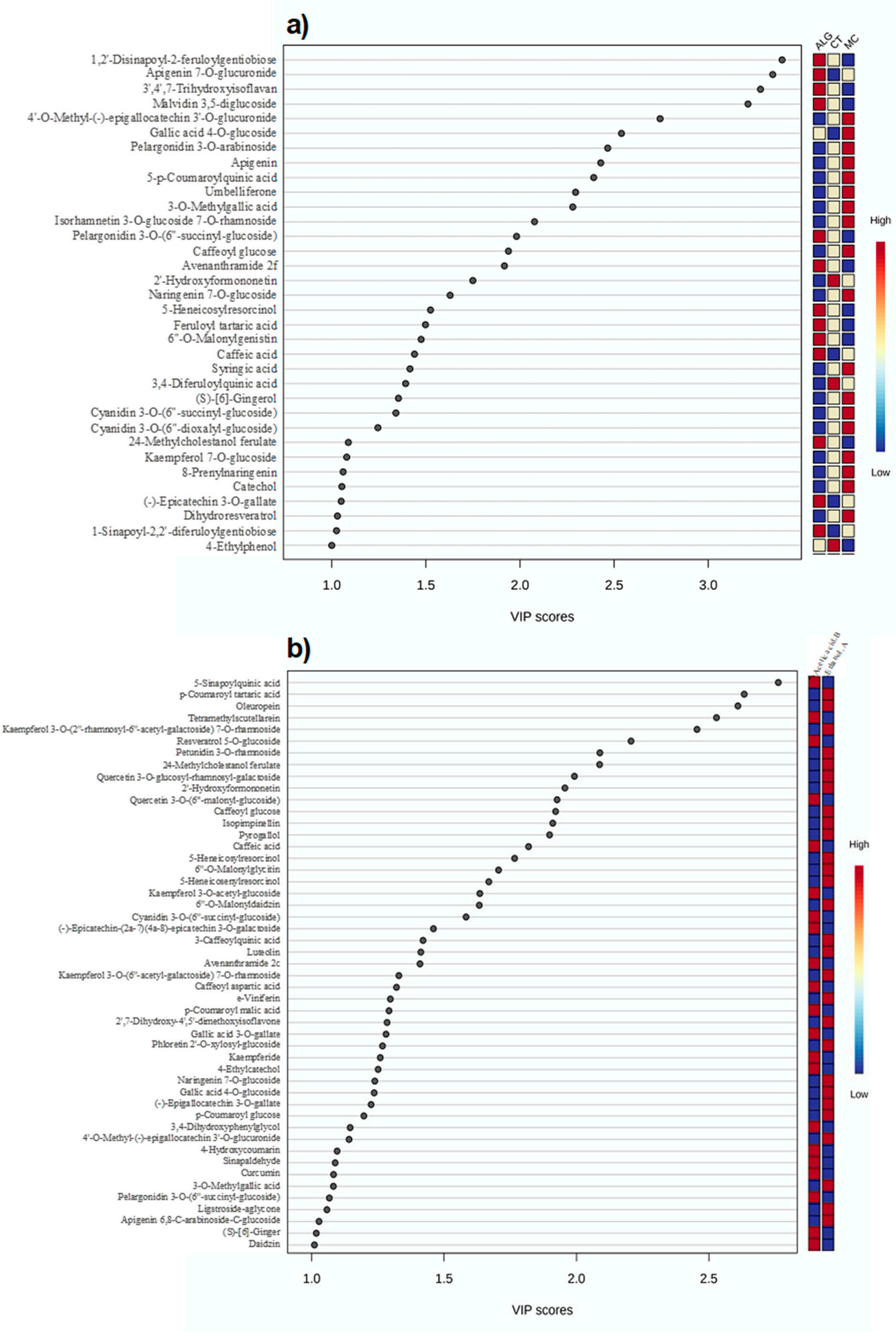

Figure S1 The VIP scores based on: a) the type of PLA film coated (ALG, CT and MC), and b) the type of food simulant used (A and B).
